# Active surveillance for antibodies confirms circulation of lyssaviruses in Palearctic bats

**DOI:** 10.1186/s12917-020-02702-y

**Published:** 2020-12-10

**Authors:** Veronika Seidlova, Jan Zukal, Jiri Brichta, Nikolay Anisimov, Grzegorz Apoznański, Hana Bandouchova, Tomáš Bartonička, Hana Berková, Alexander D. Botvinkin, Tomas Heger, Heliana Dundarova, Tomasz Kokurewicz, Petr Linhart, Oleg L. Orlov, Vladimir Piacek, Primož Presetnik, Alexandra P. Shumkina, Mikhail P. Tiunov, Frantisek Treml, Jiri Pikula

**Affiliations:** 1 Department of Ecology and Diseases of Game, Fish and Bees, University of Veterinary and Pharmaceutical Sciences Brno, Palackého tř. 1946/1, 612 42 Brno, Czech Republic; 2 Institute of Vertebrate Biology, Czech Academy of Sciences, Květná 8, 603 65 Brno, Czech Republic; 3 Department of Botany and Zoology, Masaryk University, Kotlářská 267/2, 611 37 Brno, Czech Republic; 4 Land Use and Biodiversity, International Complex Research Laboratory for Study of Climate Change, Tyumen State University, Volodarckogo 6, 625003 Tyumen, Russia; 5 Institute of Biology, Department of Vertebrate Ecology and Palaeontology, Wrocław University of Environmental and Life Sciences, Wrocław, Poland; 6 Irkutsk State Medical University, Krasnogo Vosstania street 1, 664003 Irkutsk, Russian Federation; 7 Department of Ecosystem Research, Environment Risk Assessment and Conservation Biology, Institute of Biodiversity and Ecosystem Research, Tsar Osvoboditel 1, 1000 Sofia, Bulgaria; 8 Department of Biochemistry, Ural State Medical University, Repina 3, 620014 Ekaterinburg, Russia; 9 Centre for Cartography of Fauna and Flora, Antoličičeva 1, SI-2204 Miklavž na Dravskem polju, Slovenia; 10 Western Baikal protected areas, Federal State Budgetary Institution “Zapovednoe Pribaikalye”, Baikalskaya st. 291B, 664050 Irkutsk, Russia; 11 Institute of Biology and Soil Science, Far East Branch of the Russian Academy of Sciences, Pr- t 100-letiya Vladivostoka 159, 690022 Vladivostok, Russia; 12 Department of Infectious Diseases and Microbiology, University of Veterinary and Pharmaceutical Sciences Brno, Palackého tř. 1946/1, 612 42 Brno, Czech Republic; 13 CEITEC - Central European Institute of Technology, University of Veterinary and Pharmaceutical Sciences Brno, Brno, Czech Republic

**Keywords:** Chiroptera, rabies, blood samples, seroprevalence, Europe, Siberia

## Abstract

**Background:**

Palearctic bats host a diversity of lyssaviruses, though not the classical rabies virus (RABV). As surveillance for bat rabies over the Palearctic area covering Central and Eastern Europe and Siberian regions of Russia has been irregular, we lack data on geographic and seasonal patterns of the infection.

**Results:**

To address this, we undertook serological testing, using non-lethally sampled blood, on 1027 bats of 25 species in Bulgaria, the Czech Republic, Poland, Russia and Slovenia between 2014 and 2018. The indirect enzyme-linked immunosorbent assay (ELISA) detected rabies virus anti-glycoprotein antibodies in 33 bats, giving an overall seroprevalence of 3.2%. Bat species exceeding the seroconversion threshold included *Myotis blythii*, *Myotis gracilis, Myotis petax, Myotis myotis, Murina hilgendorfi, Rhinolophus ferrumequinum* and *Vespertilio murinus.* While *Myotis* species (84.8%) and adult females (48.5%) dominated in seropositive bats, juveniles of both sexes showed no difference in seroprevalence. Higher numbers tested positive when sampled during the active season (10.5%), as compared with the hibernation period (0.9%). Bat rabies seroprevalence was significantly higher in natural habitats (4.0%) compared with synanthropic roosts (1.2%). Importantly, in 2018, we recorded 73.1% seroprevalence in a cave containing a *M. blythii* maternity colony in the Altai Krai of Russia.

**Conclusions:**

Identification of such “hotspots” of non-RABV lyssavirus circulation not only provides important information for public health protection, it can also guide research activities aimed at more in-depth bat rabies studies.

## Background

Lyssaviruses are zoonotic agents of rabies that cause fatal encephalomyelitis in mammals. Different bat species act as principal reservoirs for most lyssaviruses, though carnivores only host the type species rabies virus (RABV), which is responsible for the majority of human rabies cases [1]. While dog-bite mediated rabies can be eliminated by control measures such as obligatory animal vaccination and pre- and post-exposure prophylactic treatment, an estimated 59 000 people still die annually from rabies in underdeveloped countries [2]. In both Europe and North America, however, large-scale oral rabies vaccination campaigns have been successful in eliminating the risk of exposure to rabid wild carnivores [3–6]. Unlike New World insectivorous bats, Palearctic bats only host non-RABV lyssaviruses. Some of these are only known from single or few isolates and have been associated with either no or only sporadic human rabies cases contracted via bat bite [7–16].

Mass vaccination of reservoir populations is presently unfeasible as a control and elimination strategy for bat rabies; not only as culling of rabies-positive bat colonies runs counter to present international legislation regarding conservation of threatened species, especially EC Directive 92/43/EEC of 21 May 1992 on the Conservation of Natural Habitats and of Wild Fauna and Flora, but also as this approach may have the opposite effect on rabies epidemiology by stimulating bat dispersal [17].

Accepting that widespread endemicity and persistence of bat lyssaviruses cannot be prevented, surveillance may prove the best option for risk evaluation and public health protection [16] [18]. Two protocols presently exist for screening bat lyssavirus infection. The first is passive surveillance, which involves testing dead bats at roosting sites or close to human habitation. Laboratory submissions also include diseased bats suspected of having rabies, those that die in rescue centres or those that have injured humans [15] [19] [20]. This protocol relies on the vigilance of both the public and bat specialists in order for wildlife casualties to be reported and presented for examination. The second protocol involves active surveillance of live bats that are captured and sampled using non-lethal methods [10] [15]. Serological screening is the main method of active surveillance for bat rabies. Bat rabies surveillance activities in different countries of the Palearctic region and bat species are irregular, with decreasing intensity from west to east [10] [15] [21–26].

Bats sampled using these protocols fall into two non-overlapping groups, i.e. diseased and healthy bats, though bats from both groups may be possible carriers of lyssaviruses. Possible sources of bias, however, include the fact that readily encountered synanthropic species tend to prevail among species presented for examination and that both protocols target bats in the active season of their annual life cycle.

For several years, our group has been collecting blood for the study of host-pathogen interactions between hibernating Palearctic bats and the white-nose syndrome fungus *Pseudogymnoascus destructans* [27–37]. As such, we have hundreds of stored blood samples that provide an opportunity for screening rabies virus anti-glycoprotein antibodies. Given the varying intensity of surveillance for bat rabies over an extensive area of the Palearctic, we hypothesise that (i) circulation of lyssaviruses has previously gone undetected and, consequently, infection is underreported in some countries, and (ii) seroprevalence between summer- and winter-sampled bats will differ.

##  Results

Bats showed no clinical signs suggestive of rabies encephalomyelitis during the short period (hours) of sampling in the field or weeks of observation in the rescue centres. With 33 positive individuals, the overall rabies seroprevalence in our Palearctic collection was 3.2% (Tables 1 and 2). Bat species exceeding the seroconversion threshold of 0.123 EU/ml (derived from a calibration curve) included *R. ferrumequinum* in Bulgaria, *M. myotis* in the Czech Republic and Poland and *N. noctula* in the Czech Republic. The highest diversity of seropositive bat species, including *M. blythii*, *M. gracilis, M. petax, M. hilgendorfi* and *V. murinus*, was found in the Altai Krai of Russia. Antibody titres were mostly in the range of insufficient seroconversion, with only three bats (two *M. myotis* and one *M. blythii*) testing close to the threshold of sufficient seroconversion. A further *M. blythii* female was recorded over this threshold and had the highest measured titre of 0.805 EU/ml. Aside from the Bat’s Cave (Peshchera letuchikh myshey, Chineta reserve) in Altai Krai, Russia, where 26 bats of three species were examined and 19 individuals of two species were confirmed positive (*M. blythii* and *M. petax*) with a seroprevalence of 73.1%, seropositive bats tended to occur only sporadically. Antibody detection revealed three bat species newly identified as a seropositive, i.e. *M. hilgendorfi, M. petax* and *M. gracilis*. *Myotis* species (84.8%) and females (48.5%) were dominant among seropositive adult bats (χ^2^ = 10.07; *p* = 0.002), while the percentage of seropositive juvenile bats was the same for both sexes (χ^2^ = 0.01; *p* = 0.943). A higher percentage of bats tested positive during the active season (10.5%) than the hibernation period (0.9%), and rabies antibody prevalence was significantly higher in natural habitats (4.0%) than in synanthropic roosts (1.2%; *p* = 0.034).

**Table 1:** Summary of geographic sampling details and rabies infection status (seropositivity). BG = Bulgaria, CZ = Czech Republic, PL = Poland, RU = Russia, SLO = Slovenia, N.A. = not available

Country	Sample site	Habitat	Number examined	Species examined	Gender data (F/M)	Age data (AD/SAD/JUV)	Seropositive bats
BG	Rakitovo (Lepenitza cave)	natural	51	4	12/39	51/0/0	0
BG	Stoychovtsi (Andaka cave)	natural	26	1	16/10	26/0/0	**1**
CZ	city of Brno (building)	synanthropic	10	1	8/1 (1 N.A.)	N.A.	0
CZ	Ivanovice na Hané (building)	synanthropic	32	1	22/10	32/0/0	0
CZ	Jinačovice (rescue centre)	synanthropic	10	3	0/4 (6 N.A.)	0/4 (6 N.A.)	0
CZ	Mořina (Malá Amerika mines)	natural	41	2	11/30	32/5/4	0
CZ	Malá Morávka (Šimon and Juda mines)	natural	99	2	31/66 (2 N.A.)	71/20/6 (2 N.A.)	**1**
CZ	Adamov (Býčí skála cave)	natural	40	1	14/26	31/3/6	0
CZ	Suchdol (Kateřinská cave)	natural	68	7	12/49 (7 N.A.)	51/4/5 (8 N.A.)	0
CZ	Sloup (Sloupsko-Šošůvske caves)	natural	52	1	16/36	41/8/3	0
CZ	city of Plzeň (reconstructed building)	synanthropic	68	1	53/15	68/0/0	0
CZ	Poděbrady (rescue centre)	synanthropic	5	1	4/1	5/0/0	**1**
CZ	Vranov nad Dyjí (Ledové Sluje caves)	natural	149	12	36/84 (29 N.A.)	8/2 (139 N.A.)	**1**
PL	Nietoperek (bunker)	synanthropic	110	3	51/59	100/9/1	**2**
RU	Tigirek (Cave Jashchur)	natural	11	5	6/5	8/3	**1**
RU	Tigirek (Strashnaya cave)	natural	1	1	1/0	1/0/0	0
RU	Tigirek (netting)	natural	9	3	1/8	7/0/2	**4**
RU	Sakhyurta (Mechta cave)	natural	30	2	8/22	30/0/0	0
RU	Yelansy (Vologodskovo cave)	natural	9	1	1/8	9/0/0	0
RU	Ust-Pustynka (Peshchera letuchikh myshey)	natural	26	3	18/8	12/0/14	**19**
RU	Kozevnikovo (steppe lakes)	natural	1	1	1/0	1/0/0	**1**
RU	Spirino (Ob river)	natural	3	2	2/1	1/0/1	**2**
RU	Arakaevo (Arakaevskaya cave)	natural	10	2	5/5	10/0/0	0
RU	Pokrovskoje (Smolinskaya cave)	natural	21	3	4/17	21/0/0	0
RU	Severouralsk (Dačnaya cave)	natural	1	1	0/1	1/0/0	0
RU	Severouralsk (Komsomolskaya cave)	natural	17	3	9/7 (1 N.A.)	17/0/0	0
RU	Aramashevo (Sharkanskaya cave)	natural	25	4	12/13	25/0/0	0
RU	Severouralsk (Partizanskaya cave)	natural	14	2	12/2	14/0/0	0
RU	Cheremuchovo (Chertovo gorodishte)	natural	19	3	7/12	9/10/0	0
RU	Sergeyevka (Primorskiy Velikan cave)	natural	32	3	15/17	19/13/0	0
RU	Barsukovo (Barsukovskaya cave)	natural	20	2	7/13	14/6/0	0
SLO	Lovrenc na Pohorju (church Devica Maria)	synanthropic	17	1	10/7	17/0/0	0
**Total**			**1027**	**26**	**405/576 (46 N.A.)**	**737/83/41 (165)**	**33**

**Table 2:** Characteristics of rabies seropositive bats. BG = Bulgaria, CZ = Czech Republic, PL = Poland, RU = Russia, SLO = Slovenia, N.A. = not available

Bat species	Country	Sample site	Sampling date	Gender	Age	Titre (EU/ml)
*Rhinolophus ferrumequinum*	BG	Rakitovo (Lepenitza cave)	01.04.2017	female	adult	0.141
*Myotis myotis*	CZ	Malá Morávka (Šimon and Juda mines)	15.04.2014	N.A.	N.A.	0.499
*Myotis myotis*	CZ	Vranov nad Dyjí (Ledové Sluje caves)	10.04.2015	male	adult	0.147
*Nyctalus noctula*	CZ	Poděbrady (rescue centre)	07.12.2015	female	adult	0.191
*Myotis myotis*	PL	Nietoperek (bunker)	19.03.2016	female	sub-adult	0.125
*Myotis myotis*	PL	Nietoperek (bunker)	20.03.2016	female	adult	0.473
*Murina hilgendorfi*	RU	Tigirek (Cave Jashchur)	15.04.2018	male	adult	0.440
*Vespertilio murinus*	RU	Kozevnikovo (steppe lakes)	05.08.2018	female	adult	0.235
*Vespertilio murinus*	RU	Spirino (Ob river)	07.08.2018	female	juvenile	0.156
*Myotis gracilis*	RU	Spirino (Ob river)	07.08.2018	male	adult	0.271
*Myotis blythii*	RU	Ust-Pustynka (Peshchera letuchikh myshey)	13.08.2018	female	adult	0.136
*Myotis blythii*	RU	Ust-Pustynka (Peshchera letuchikh myshey)	13.08.2018	female	adult	0.123
*Myotis blythii*	RU	Ust-Pustynka (Peshchera letuchikh myshey)	13.08.2018	male	juvenile	0.321
*Myotis blythii*	RU	Ust-Pustynka (Peshchera letuchikh myshey)	13.08.2018	female	juvenile	0.176
*Myotis blythii*	RU	Ust-Pustynka (Peshchera letuchikh myshey)	13.08.2018	female	adult	0.370
*Myotis blythii*	RU	Ust-Pustynka (Peshchera letuchikh myshey)	13.08.2018	male	juvenile	0.174
*Myotis blythii*	RU	Ust-Pustynka (Peshchera letuchikh myshey)	13.08.2018	female	adult	0.805
*Myotis blythii*	RU	Ust-Pustynka (Peshchera letuchikh myshey)	13.08.2018	female	adult	0.163
*Myotis blythii*	RU	Ust-Pustynka (Peshchera letuchikh myshey)	13.08.2018	female	adult	0.233
*Myotis blythii*	RU	Ust-Pustynka (Peshchera letuchikh myshey)	13.08.2018	female	adult	0.198
*Myotis blythii*	RU	Ust-Pustynka (Peshchera letuchikh myshey)	13.08.2018	female	adult	0.451
*Myotis blythii*	RU	Ust-Pustynka (Peshchera letuchikh myshey)	13.08.2018	female	juvenile	0.169
*Myotis blythii*	RU	Ust-Pustynka (Peshchera letuchikh myshey)	13.08.2018	female	adult	0.328
*Myotis petax*	RU	Tigirek (netting)	10.08.2018	male	adult	0.145
*Myotis petax*	RU	Tigirek (netting)	10.08.2018	male	adult	0.139
*Myotis petax*	RU	Tigirek (netting)	12.08.2018	male	juvenile	0.235
*Myotis petax*	RU	Tigirek (netting)	12.08.2018	male	juvenile	0.163
*Myotis petax*	RU	Ust-Pustynka (Peshchera letuchikh myshey)	13.08.2018	male	juvenile	0.323
*Myotis petax*	RU	Ust-Pustynka (Peshchera letuchikh myshey)	13.08.2018	female	adult	0.229
*Myotis petax*	RU	Ust-Pustynka (Peshchera letuchikh myshey)	13.08.2018	male	juvenile	0.139
*Myotis petax*	RU	Ust-Pustynka (Peshchera letuchikh myshey)	13.08.2018	female	adult	0.187
*Myotis petax*	RU	Ust-Pustynka (Peshchera letuchikh myshey)	13.08.2018	male	juvenile	0.277
*Myotis petax*	RU	Ust-Pustynka (Peshchera letuchikh myshey)	13.08.2018	female	adult	0.180

##  Discussion

In this study, we were able to extend active surveillance of bat rabies to selected countries of Central and Eastern Europe and eastern Palearctic (Siberian and Far East) regions of Russia. Despite the fact that the bats were primarily sampled for a different purpose, the seroprevalence data obtained suggests both ongoing exposure of Palearctic bats to lyssavirus circulation and spatial and temporal variation in bat rabies infection.

Rabies invariably proves fatal for mammals once clinical signs develop. On the other hand, much is still poorly understood regarding bats infected with non-RABV lyssaviruses, e.g. persistence in natural hosts, pathogenesis and epidemiology [15] [38]. For example, some bats survive natural exposure to non-RABV lyssaviruses and go on to produce post-infection antibodies [15] [21–24] [39], and yet little is known about antiviral functions of the chiropteran immune system [40–42]. *In vitro* experiments with brain cell lines derived from *M. myotis*, however, suggest that high up-regulation of pattern recognition receptors and a vigorous interferon-mediated response are invoked to control lyssavirus infection [43].

Lyssaviruses identified to circulate in bat species of the studied geographic region include European bat lyssavirus type 1 and 2, Bokeloh bat lyssavirus, Lleida bat lyssavirus, West Caucasian bat lyssavirus, and lyssaviruses Aravan, Khujand and Irkut [7] [9] [13] [14] [16]. The commercial ELISA kit used in this study employes RABV glycoprotein to detect anti-rabies antibodies. It is a multi-species assay using protein A (a surface molecule of *Staphylococcus aureus* binding to the Fc portion of immunoglobulins from a large number of animal species) coupled with peroxidase as an enzymatic conjugate. Detection of cross-reactivity between sera against divergent members of the lyssavirus genus was recorded [1] [44] [45] [46]. Though it is for the classical RABV detection, using this kit has proved advantageous when testing samples from mixed bat populations collected over an extensive area with several possible non-RABV lyssaviruses circulating. However, bats exposed to phylogenetically distant lyssaviruses may not have been detected; meaning that rabies prevalence is probably underestimated in our study. ELISA including RABV as one of antigens has already been used to screen bats for rabies in Old World bats [47]. Interestingly, approximately 11% of 789 bats sampled in Northern Vietnam had neutralizing antibodies against RABV [48]. As ELISA detects binding antibodies to rabies glycoprotein G, it may differ in sensitivity and susceptibility when compared with standard virus neutralization assays, i.e. the fluorescent antibody virus neutralization (FAVN) or the rapid fluorescent focus inhibition (RFFIT) [47]. The body mass of Palearctic bat species limits the blood volume available for non-lethal collection. It is therefore very difficult to yield enough blood for screening a range of different lyssaviruses using FAVN or RFFIT reactions.

Though there are no validated standards for bat sera [47] and the study by Wasniewski et al. (2014) [49] suggested decreasing the cut-off threshold (ELISA tests) to increase the test sensitivity for domestic carnivores and wildlife, our data show that ELISA could be used as a quick screening tool for bat exposure to lyssaviruses. However, further validation and effectiveness testing of the method for bat samples in combination with reference neutralization tests will be necessary. Problems associated with diagnostic sensitivity, specificity, sample volume requirements and detection of multiple lyssaviruses could be resolved by development of a bead-based multiplex immunoassay [50] for bat rabies screening.

To our knowledge, this is the first active surveillance study to report *M. hilgendorfi, M. petax* and *M. gracilis* as rabies seropositive, though *M. hilgendorfi* has previously been associated with rabies as *M. leucogaster*, recorded as positive in the 2002 Irkut lyssavirus isolation [7], is now considered to be *M. hilgendorfi* in the Baikal Lake region. In addition, seropositivity of *M. petax* and *M. gracilis*, sibling species to *M. daubentonii* and *M. brandtii*, respectively, matches the documented association between *Myotis* bat species and European bat lyssavirus type 2 (EBLV-2) [10] [15].

The overall rabies seroprevalence in our Palearctic collection was 3.2% and when we remove seropositive bat colony (Altai Krai, Russia), seroprevalence in the Europe decreases (2.0%).

**Fig. 1:**
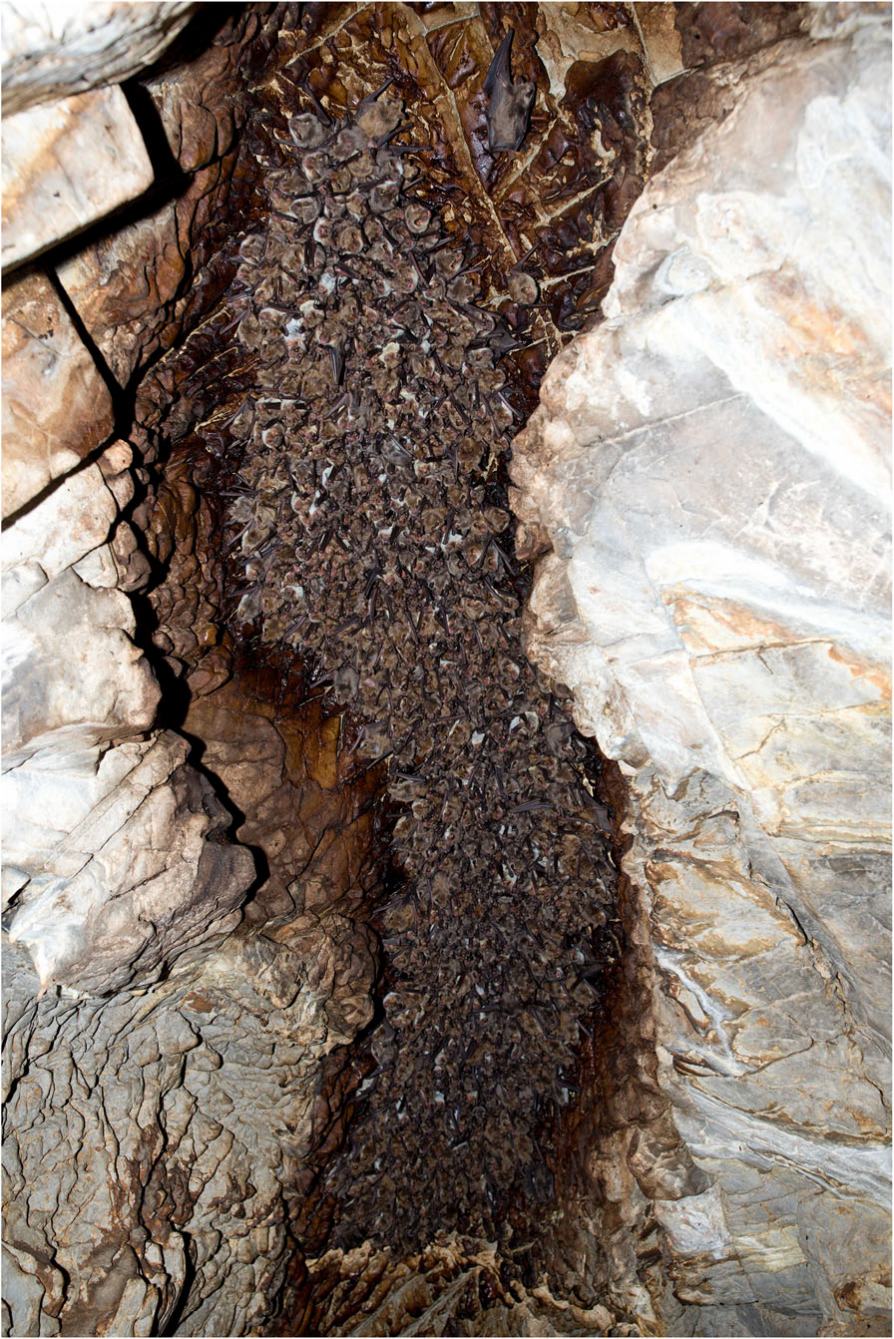
Bats roosting in the Peshchera letuchikh myshey (Chineta reserve, Altai Krai, Russia). This *Myotis blythii* maternity colony comprises approximately 1400 bats, including females and young-of-the-year. As shown, individual bats are tightly clustered during the nursery period, allowing for high contact intensity

*Myotis* species represented the majority (84.8%) of seropositive records in this study (Table 2), though one seropositive sampling site stood out from all others with a seroprevalence level of 73.1%. While the two species confirmed positive (*M. blythii* and *M. petax*) occupied different roosts in the Bat’s cave (Peshchera letuchikh myshey, Chineta reserve, Altai Krai, Russia), seropositivity indicated possible interspecies lyssavirus circulation. Large, tightly clustered maternity colonies of *M. blythii* (Fig. 1), allow for high contact intensity and may be the driver of lyssavirus circulation at this site. An analogy can be drawn between this site and serological status associated with circulation of European bat lyssavirus type 1 (EBLV-1) in maternity colonies of *Eptesicus serotinus* in France [24] and *M. myotis* in Spain [21] [39] *Myotis petax* and *Myotis gracilis* are examples of cryptic bat species paired with Palearctic bat rabies positive species, i.e. *Myotis daubentonii* and *Myotis brandtii* [51]. Two boreal species, European *Myotis brandtii* and Asian *Myotis gracilis* which are closely related, belong to the New World clade. These morphologically almost indistinguishable species were long considered as synonyms of *Myotis mystacinus*. On the contrary, morphologically very similar Palearctic species, Asian *Myotis petax* and European *Myotis daubentonii*, appear distantly related within Old World *Myotis.* They were long considered a single species with the Western Palearctic *M. daubentonii*, but multiple lines of evidence [52] [53] clearly show that they are not even sister-species [51]. Even though the evolutionary history of these pairs is different, we may expect that the potential for harbouring pathogens will be similar, including the cross-species positivity.

Detection of cross-reactive antibodies using ELISA provides no clue to the identity of circulating lyssaviruses, however, other than it belongs to phylogroup I [1]. Interestingly, in 1991, Aravan lyssavirus (ARAV) was isolated from a male *M. blythii* in Kyrgyzstan (Central Asia) [9], suggesting that bats roosting in this cave need to be sampled for identification of circulating viruses.

The higher percentage of seropositive adult females (48.5%) in this study corresponds with the findings of Picard-Meyer et al. (2011) and Robardet et al. (2017), suggesting that females may act as a suitable indicator of lyssavirus circulation in bat populations. In comparison, the more solitary behaviour of male bats results in a lower probability of exposure to lyssaviruses. Interestingly, male vampire bats were shown to contribute disproportionately to rabies spatial spread [54]. Our finding of no difference in seropositive prevalence between the sexes in juvenile bats (ca. two months) is most likely due to an equal chance of exposure during the nursery period or the passing of maternally acquired antibodies to both sexes.

## Conclusions

Our results suggest that active surveillance of bat rabies is more efficient (i.e. providing higher rate of positive findings) when samples are collected during the bat’s active season, rather than during the hibernation period. While both innate and adaptive immune functions are suppressed during hibernation [41] [42] [55], the rate of infection transmission contacts is also reduced. Colony size, species richness, high mobility and the social behaviour of bats during the active season when summer colonies are formed and swarming occurs provide higher chances for pathogen transmission between individuals [18] [21] [56]. Likewise, during the active season the bats´ immune system better responds to antigenic stimulation increasing antibody production and chances to detect seropositive animals in a population in which lyssaviruses circulate. Further, it would appear that human-bat contact in natural habitats poses a higher exposure risk, which is of importance as regards public health. Obviously, there is a considerable difference in risk of exposure for the general public and bat specialists, researchers and wildlife rehabilitators. The risks, however, cannot be generalised, meaning that geographic, species diversity, population density and annual life cycle issues have to be considered separately in each case.

##  Methods

### Material collection

Blood samples were taken from 1027 bats of 25 different species in Bulgaria (n = 77), the Czech Republic (n = 574), Poland (n = 110), Russia (n = 249) and Slovenia (n = 17) between 2014 and 2018. Every bat was sampled just one time. Using morphological traits and/or sequencing of the mitochondrial gene for cytochrome *b* (*mtcyb*), bat species were identified as *Barbastella barbastellus* (*n* = 11), *Eptesicus nilssonii* (*n* = 26), *Eptesicus serotinus* (*n* = 3), *Miniopterus schreibersii* (*n* = 44), *Murina hilgendorfi* (*n* = 42), *Myotis alcathoe* (*n* = 2), *Myotis bechsteinii* (*n* = 18), *Myotis blythii* (*n* = 20), *Myotis bombinus* (*n* = 2), *Myotis brandtii* (*n* = 33), *Myotis dasycneme* (*n* = 52), *Myotis daubentonii* (*n* = 86), *Myotis emarginatus* (*n* = 20), *Myotis gracilis* (*n* = 36), *Myotis myotis* (*n* = 328), *Myotis mystacinus* (*n* = 3), *Myotis naterreri* (*n* = 47), *Myotis petax* (*n* = 27), *Nyctalus noctula* (*n* = 118), *Pipistrellus pipistrellus* (*n* = 3), *Plecotus auritus* (*n* = 50), *Plecotus ognevi* (*n* = 4), *Rhinolophus euryale* (*n* = 13), *Rhinolophus ferrumequinum* (*n* = 35) and *Vespertilio murinus* (*n* = 6) [31] [34] The external genitalia of each bat was inspected for gender determination and age was estimated based on epiphyseal ossification of the thoracic limb fingers and teeth abrasion [57]. Bats were sampled at underground hibernacula, summer colonies, swarming sites and rescue centres [31] [32] [58], with bats captured during the annual life cycle active season and the hibernation period numbering 247 and 780 specimens, respectively. For further detail on sampling site, habitat type, number of specimens examined, species and gender, see Tables 1 and 2.

Every attempt was made to minimise the impact of disturbance, handling stress and sampling procedure duration. Bats emerging from hibernacula, roosts or swarming sites were captured using mist-nets, with active bats sampled immediately following capture and released as soon as possible thereafter. For those bats in torpor, we allowed a re-warming period of about 60 minutes to allow for efficient blood collection. In all cases, the skin surface was first disinfected with alcohol, the uropatagial vessel was then lanced using a sterile needle and a 15 µl blood sample was taken using a pipette tip. A drop of tissue surgical glue (Surgibond, SMI AG, Belgium) was then applied to seal the lacerated skin and prevent further bleeding. Prior to release, all bats were provided with 5% glucose for rapid replenishment of energy and fluids.

## Detection of rabies virus anti-glycoprotein antibodies

Blood samples were examined using the indirect enzyme-linked immunosorbent assay (ELISA) (PLATELIA™ RABIES II kit, BIO-RAD, France), which allows for qualitative detection and titration of IgG antibodies against the rabies virus glycoprotein G in animal serum. Detailed data concerning the antigen, reagents and procedures used in the Platelia kit can be found in [59]. In general, we followed the manufacturer’s recommendations and used the supplied positive controls and quantification standards; the only modification to the recommended protocol being mixing of the whole blood sample with sample diluent immediately after blood collection and subsequent centrifugation to remove formed elements. Samples were then kept frozen (-20 °C) until serological analysis. Prior to testing bat samples we performed in-house validation by comparison of results obtained using the commercial PLATELIA RABIES II kit and the reference FAVN test. Positive (post-vaccination) and negative paired whole blood and serum samples of domestic and wild carnivores (*n* = 30) were used for this purpose, providing 100% specificity for both sample types and insignificant differences in sensitivity (88 and 94%, respectively). A set of these carnivore samples was included later in each run with bat samples and, together with positive and negative controls supplied with the kit, used to construct a calibration curve for conversion of measured optical density values into titres as well as to derive the classification thresholds. The following threshold levels were applied for titre interpretation: (a) negative results representing undetectable seroconversion (< 0.123 Equivalent Units/ml), (b) positive results with insufficient seroconversion (0.123 to 0.5 EU/ml), and (c) positive results with sufficient seroconversion (> 0.5 EU/ml). Percentage bat seropositivity in different habitat types was compared by testing the difference between two proportions. The Chi-square test with Yate’s correction was used to detect seropositivity patterns by age and sex.

## Data Availability

All data needed to evaluate the conclusions are present in the paper. The raw datasets used and/or analysed during the current study available from the corresponding author on reasonable request.

## Abbreviations

EBLV-1: European bat lyssavirus type 1
EBLV-2: European bat lyssavirus type 2
ELISA: Enzyme-linked immunosorbent assay
RABV: Rabies virus
